# Preparation of Mn-Based Selective Catalytic Reduction Catalysts by Three Methods and Optimization of Process Conditions

**DOI:** 10.1371/journal.pone.0073237

**Published:** 2013-09-04

**Authors:** Yi Xing, Chen Hong, Bei Cheng, Kun Zhang

**Affiliations:** 1 Key Laboratory of Educational Ministry for High Efficient Mining and Safety in Metal Mine, University of Science and Technology Beijing, Beijing, China; 2 Department of Environmental Engineering, University of Science and Technology Beijing, Beijing, China; Queen's University Belfast, United Kingdom

## Abstract

Mn-based catalysts enable high NO_*x*_ conversion in the selective catalytic reduction of NO_*x*_ with NH_3_. Three catalyst-production methods, namely, co-precipitation, impregnation, and sol-gel, were used in this study to determine the optimum method and parameters. The maximum catalytic activity was found for the catalyst prepared by sol-gel with a 0.5 Mn/Ti ratio. The denitrification efficiency using this catalyst was >90%, which was higher than those of catalysts prepared by the two other methods. The critical temperature of catalytic activity was 353 K. The optimum manganese acetate concentration and weathering time were 0.10 mol and 24 h, respectively. The gas hourly space velocity and O_2_ concentration were determined to be 12000 h^-1^ and 3%, respectively.

## Introduction

Rapid economic development in China is increasing the demand for electricity, leading to elevated total thermal power plant NO_*x*_ emissions that significantly harm the environment and human life. The selective catalytic reduction (SCR) of NO_*x*_ with NH_3_ is one of the most effective methods of NO_*x*_ removal [1]. The key to success of the entire SCR system is catalyst design and selection as determined by the flue gas conditions and components. Many catalyst systems such as V_2_O_5_–WO_3_/TiO_2_, Mn/TiO_2_, CeO_2_/TiO_2_ [2], and many metal composite oxide catalysts have also been extensively investigated [3–5].

Low-temperature catalytic processes usually ranging from 393 K to 573 K or lower are extensively studied. Meanwhile, manganese oxide is well known for its high activity in the SCR of NO_*x*_ at low temperatures [6,7]. In recent years, manganese-based catalysts have attracted significant attention because of their high activity in various reactions such as the direct decomposition of nitric oxide, CO oxidation, CH_4_ oxidation, and total oxidation of volatile organic compounds [8–10].

Pena et al. [11] compared the catalytic properties of metal oxides and the reaction selectivity. They found that different active materials have different catalytic surface properties when loaded on TiO_2_, and thus significantly affect the reaction. Although these metal oxides all have high catalytic activity above 473 K, the catalytic activity differs at low temperatures. For example, MnO_*x*_ can achieve high catalytic activity at about 373 K, but other metal oxides only exhibit about 20% NO removal rate at this temperature. When the temperature is increased to 373-473 K, NO removal efficiency gradually increases.

SCR catalysts are mainly prepared by three methods, namely, co-precipitation, impregnation method, and sol-gel methods [12]. Few studies have been conducted on the sol-gel method for preparing SCR at low temperatures. However, the sol-gel method is commonly used effectively to prepare catalysts for contaminant removal. Therefore, the sol-gel method can be an effective way of preparing highly active catalysts for low-temperature SCR [13]. The sol-gel method can promote the directional adsorption of ions and decrease the temperature of crystal-phase formation, resulting in uniform, stable, and anti-sintering catalysts [14]. Thus, this method is promising for low-temperature SCR catalyst preparation. Yamazoe [15] prepared WO_3_/TiO_2_ catalysts and achieved the optimum activity.

The present study aimed to compare the performances of Mn/TiO_2_ (s) catalysts prepared by co-precipitation, impregnation, and sol-gel methods in NO_*x*_ removal at low temperatures to determine the optimum method [16]. The reaction conditions (manganese concentration, weathering time, and temperature) were also investigated because they crucially affect the catalytic activity. Apart from the production parameters, the operational parameters (gas hourly space velocity (GHSV) and O_2_ concentration) were investigated as well because different catalysts require different GHSVs and O_2_ influences adsorption and catalysis [17]. After selecting the optimum method of preparing low-temperature NO_*x*_-removal catalysts, single-factor experiments were performed to determine the optimum production and operational parameters.

## Experimental

### 2.1: Catalyst preparation method

#### 2.1.1: Co-precipitation method

Catalysts were prepared by initially dissolving manganese acetate and nitrate titanium in 200 mL of deionized water. Then, NH_3_·H_2_O (1:1) solution was added dropwise at 368 K with water refluxing until no solid precipitated. The solution was constantly stirred at 368 K for 2 h, centrifuged, and washed with deionized water five times. The obtained solid was dried for 24 h in 378 K, ground, sieved (60–100 mesh), and calcined for 6 h in air in a furnace. The produced catalyst is hereafter denoted as Mn(*z*)/TiO_2_(c), where *z* is the molar ratio of Mn/Ti and c means co-precipitation.

#### 2.1.2: Impregnation method

Cellular ceramic was used as a carrier for manganese acetate and TiO_2_. Manganese acetate was dissolved in 20 mL of deionized water and mixed with 4 g of TiO_2_. Then, NH_3_·H_2_O (1:1) was added to the mixture until no brown substance precipitated. The mixture was centrifuged, washed with deionized water three times, and dried in a 378 K oven for 24 h. The obtained porous solids were then ground, sieved (60–100 mesh), and calcined for 6 h in air in a furnace.

#### 2.1.3: Sol-gel method

Titanate, n-butyl, and manganese acetate were used in this method. About 0.1 mol tetrabutyl titanate and 0.15 mol ethanol were mixed in a beaker and vigorously stirred. Then, 0.1 mol of manganese acetate was dissolved in a small amount of deionized water in another beaker. The manganese acetate solution was slowly added dropwise to a beaker containing esters under constant stirring. The mixture was further stirred for 30 min until bright yellow jellies were observed. The jellies were left to stand for 24 h and dried for another 24 h at 373 K to obtain yellow solids. The solids were ground to powder, sieved (80–100 mesh), and calcined for 6 h at 500 °C in a furnace. Finally, dark brown powders were obtained.

### 2.2: Experimental apparatus

**Figure 1:**
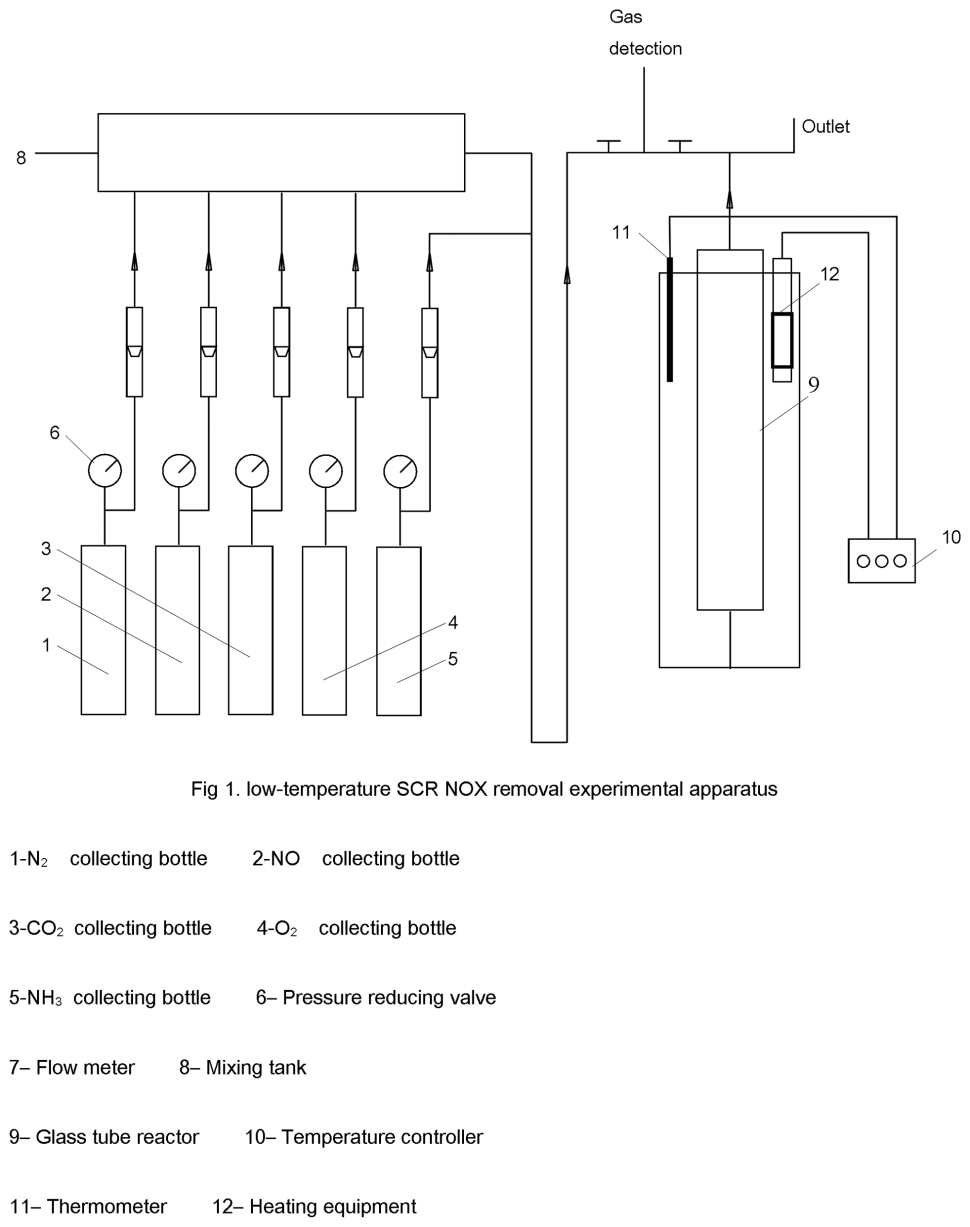
Low-temperature SCR NO_x_ removal experimental apparatus.

The low-temperature SCR NO_*x*_-removal experimental apparatus is shown in Figure 1. The SCR reaction occurred in a glass tube (0.8 cm in diameter) from 353 K to 393 K. The reaction was divided into integral and differential reactions (only the integral reaction is given focus in this study). In the integral reaction, 1 g of catalyst was added to the reactor and 1.5 g of catalyst was added to the differential reaction. The reaction temperature was controlled by a sleeve-type heater. N_2_ was added to the system before and after reaction to avoid the effect of residual gases. At the beginning of reaction, adsorption saturation experiments were conducted. When the NO_*x*_ concentrations of the reactor inlet and outlet were balanced, NH_3_ was added to initiate the reaction.

The most typical conditions of the catalytic reaction were as follows: total gas flow rate, 680 mL/min (N_2_ and CO_2_ were the carrier gases); N_2_ flow rate, 430 ml/min; CO_2_ flow rate, 50 mL/min; NO flow rate, 40 mL/min; O_2_ flow rate, 80 mL/min; NH_3_ flow rate, 80 mL/min; inlet NO_*x*_ concentration, 1000-1100 ppm; and space velocity, 30 000 h^-1^.

### 2.3: Analytical methods

An NO–NO_2_–NO_*x*_ Analyzer (Testo 350 M/XL) was used for gas detection. Total NO_*x*_ was determined based on the NO and NO_2_ concentrations.

## Results and Discussion

### 3.1: Effect of preparation methods on catalytic activity

The effect of the Mn/Ti ratio was determined because this ratio is regarded as one of the main factors determining the catalytic activity. In this experiment, the Mn/Ti ratios used for reaction were varied (0.2, 0.3, 0.4, 0.5, and 0.6) by adding manganese acetate.

**Figure 2:**
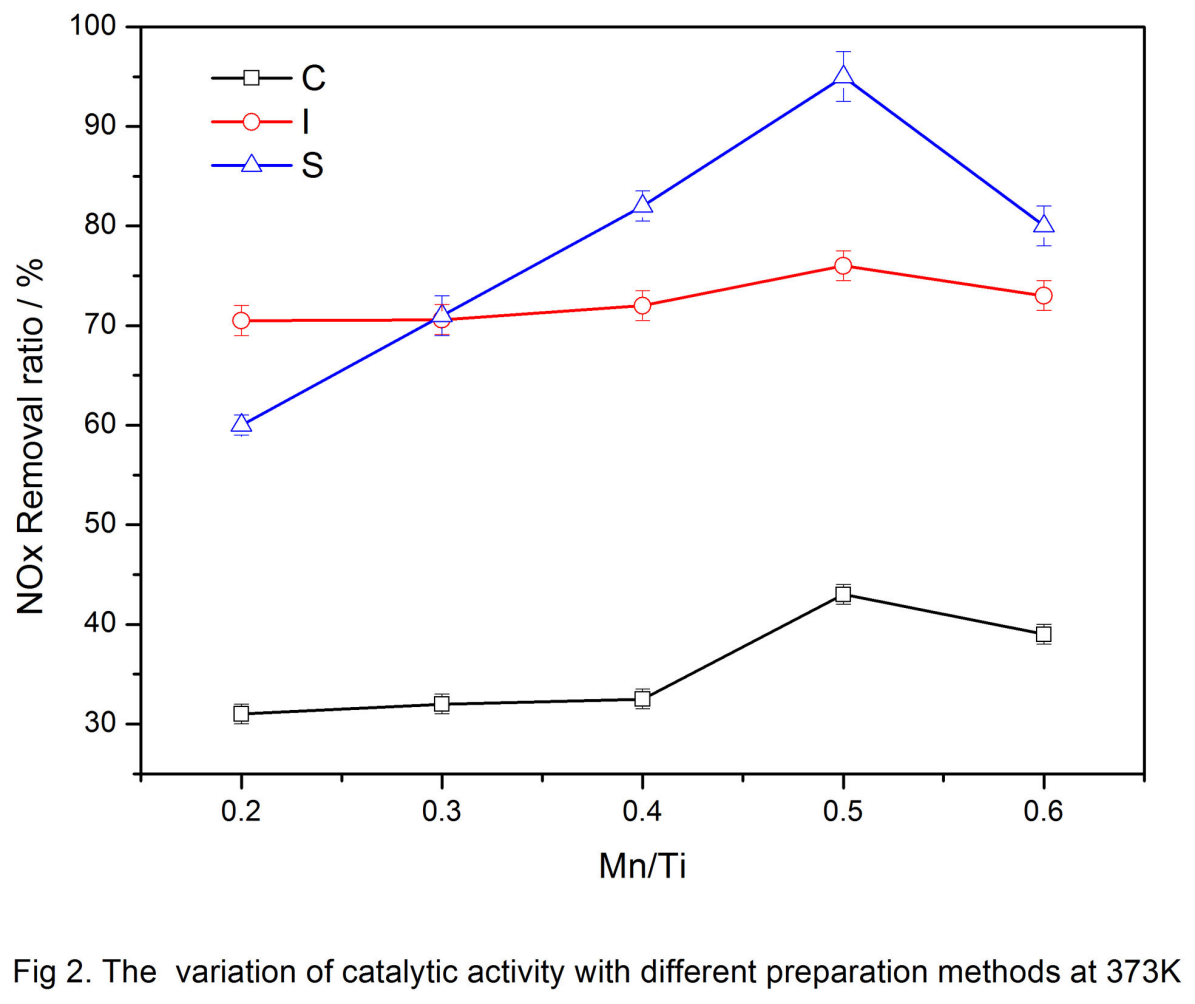
The variation of catalytic activity with different preparation methods at 373K.

Catalysts were prepared by co-precipitation, impregnation, and sol-gel methods. Catalytic activity was measured by the NO_*x*_ removal rate at 373 K. The results are shown in Figure 2, where C, I, and S denote co-precipitation, impregnation, and sol-gel, respectively.

The co-precipitation method showed a low NO_*x*_ removal rate, with the highest rate being only 45%. The load capacity of Mn had little effect on catalytic activity, which agreed with the findings of. Yang [18]. When the Mn/Ti ratio was 0.5, the NO_*x*_ removal rate reached the highest at 373 K but did not meet the desired effects. Thus, this ratio was not selected.

In the impregnation method, the NO_*x*_ removal rate was high and the load capacity of Mn had the least effect on catalytic activity. The NO_*x*_ removal rate remained at about 70%.

In the sol-gel method, the load capacity of Mn significantly affected the catalytic activity. When the Mn/Ti ratio was <0.5, the catalytic activity rapidly increased with increased load capacity of Mn, as shown in Figure 2. This trend was due to a higher concentration of the active phase on the catalyst surface. The catalyst achieved the highest activity when the Mn/Ti ratio was 0.5, beyond which NO_*x*_ removal did not increase and instead decreased.

Among the catalysts prepared by three different preparation methods, the sol-gel-prepared catalyst was the most sensitive to the Mn load capacity, achieving >90% NO_*x*_ removal when the Mn/Ti ratio was 0.5. The impregnation-prepared catalyst was less sensitive to the Mn load capacity, but the NO_*x*_ removal rate was maintained at about 70% because of poor catalytic activity. When the Mn/Ti ratio was 0.5, the co-precipitation-prepared catalyst also achieved the highest NO_*x*_ removal rate, but was below 50%. Thus, the sol-gel method was deemed as the optimum method.

### 3.2: Influence of reaction temperature on catalytic activity

The reaction temperature significantly affects the catalytic activity. Generally, the appropriate reaction temperature ranges from 353 K to 393 K. The reaction temperature also affects the catalyst adsorption process. Therefore, the optimum reaction temperature was determined by the NO_*x*_ removal efficiency and the effective time of catalyst. A lower gas temperature was found to decrease the catalytic activity, thereby reducing the NO_*x*_ removal efficiency. A high gas temperature initiated the reaction between NH_3_ and O_2_, which increased the NO_*x*_ concentration in the flue gas, thereby offsetting the denitrification effect. The reaction rate and catalytic activity also changed because of different flue gas temperatures.

The reaction temperature caused a mutual conversion reaction among gases. The reaction temperature also affected the catalyst life and adsorption effect mainly because the pore structure of the catalyst surface varied at different reaction temperatures. Furthermore, impurities generated at different temperatures blocked the catalyst pore structure, resulting in lower catalytic activity and shorter catalyst effective time.

**Figure 3:**
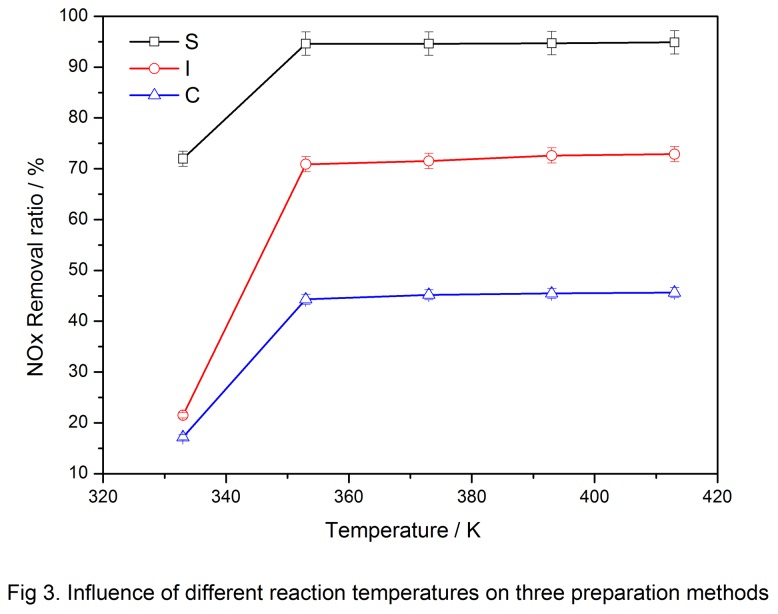
Influence of different reaction temperatures on three preparation methods.

Five temperatures ranging from 333 K to 413 K were selected to investigate the optimum reaction temperature, and the results are shown in Figure 3.

Figure 3 shows that the sol-gel-prepared catalyst had the highest NO_*x*_ removal rate within a low temperature range (330 K to 380 K), and >90% of NO_*x*_ was removed at 353 K. When the NO_*x*_ removal rate was <75% for the impregnation and co-precipitation methods, the removal rate was <50%. In industrial practice, denitrification generally occurs after desulfurization; thus, the flue gas temperature after denitrification was lower than 423 K and the catalytic activity below 423 K was the most critical. Among the three samples, the activity of the sol-gel-prepared catalyst was significantly higher than those of the other two catalysts. Moreover, the sol-gel-prepared catalyst had excellently retained high activity. Figure 3 shows that the critical temperature of catalytic activity was 353 K. The activity of the three samples was basically unchanged after the temperature reached 353 K, and the NO_*x*_ removal rate of the sol-gel-prepared catalyst remained >90%.

### 3.3: Influence of manganese acetate concentration on catalytic activity

**Figure 4:**
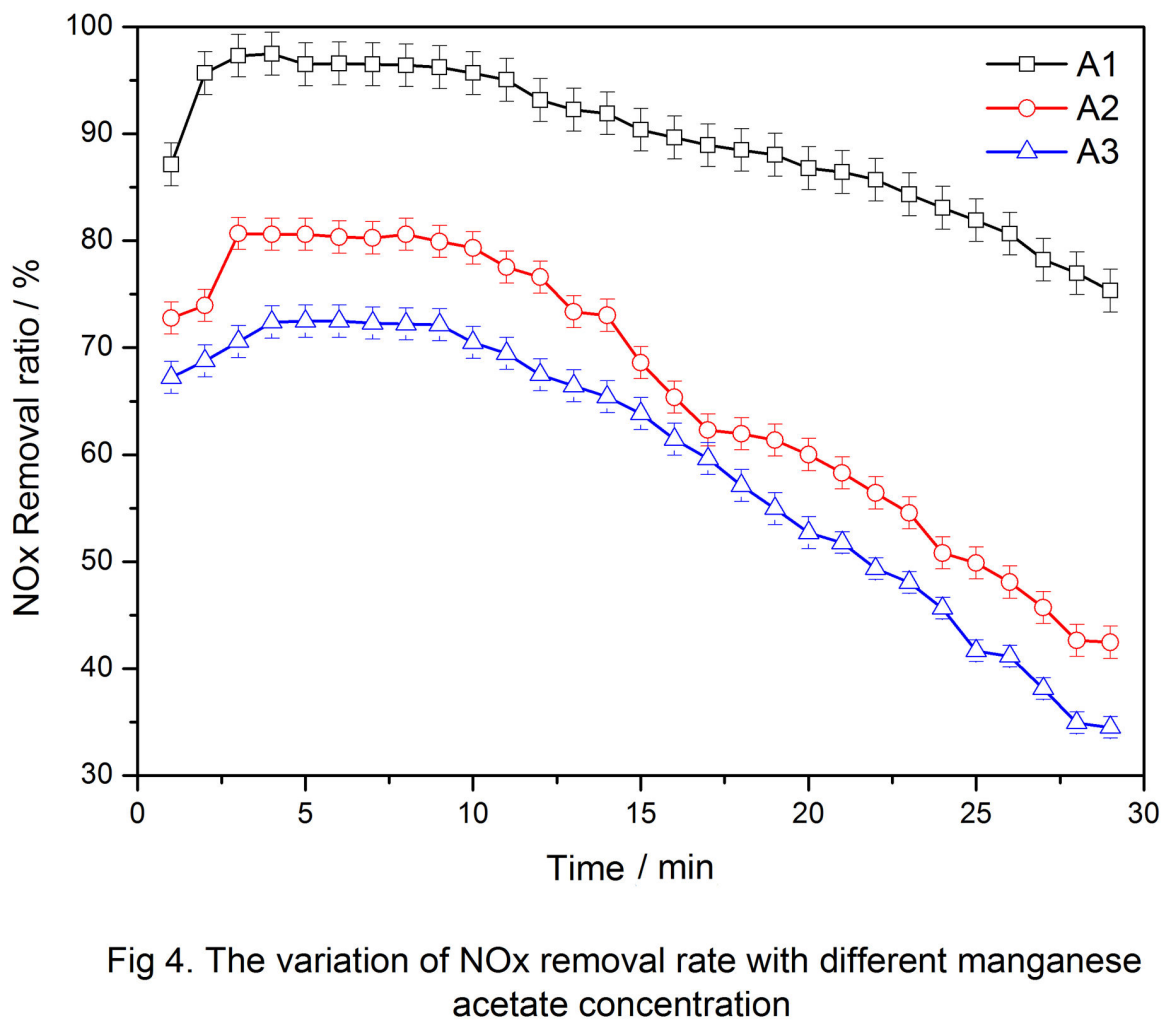
The variation of NO_x_ removal rate with different manganese acetate concentration.

In this experiment, 0.10, 0.20, and 0.4 mol manganese acetate were added to prepare catalysts denoted as A1, A2, and A3, respectively. Catalysts were prepared by the sol-gel method as discussed in the subsection “Sol-gel method.” About 1.5 g of A1, A2, and A3 were added to the flue gas simulation SCR reactor, and then mixed gases were added as mentioned in 2.2 when the temperature was stable at 353 K. The inlet NO_*x*_ concentration was recorded when the outlet NO_*x*_ concentration stabilized. Then, NH_3_ was added and the outlet NO_*x*_ concentration was recorded every 1 min. Figure 4 shows the NO_*x*_ concentration of the three samples in reaction for different times.

Figure 4 shows that the manganese acetate concentration of 0.10 mol had the optimum catalytic activity with >95% NO_*x*_ removal rate. With further increased manganese acetate concentration, the catalytic activity gradually decreased. After 25 min, the catalyst almost had no effect (removal efficiency < 80%). The catalytic activity also decreased with increased manganese acetate concentration, and the NO_*x*_ removal rate was only 70% at 0.40 mol of manganese acetate. The results indicated that the Mn concentration significantly affected the NO_*x*_ removal rate, and 0.10 mol of manganese acetate achieved the optimum catalytic activity.

### 3.4: Influence of weathering time on catalytic activity

Catalytic activity is also significantly affected by weathering time. Weathering is the length of time catalysts are dried in air.

Jiang Boqiong [16] reported that weathering time affects the compactness of the catalyst crystal structure. Therefore, the weathering times (24, 48, and 72 h) were varied in the experiment while other reaction conditions were fixed based on the optimum combination (0.1 mol of manganese acetate, particle size of 80 to 100 mesh, and 2 h of calcination time). The prepared catalysts are denoted as B1, B2, and B3.

**Figure 5:**
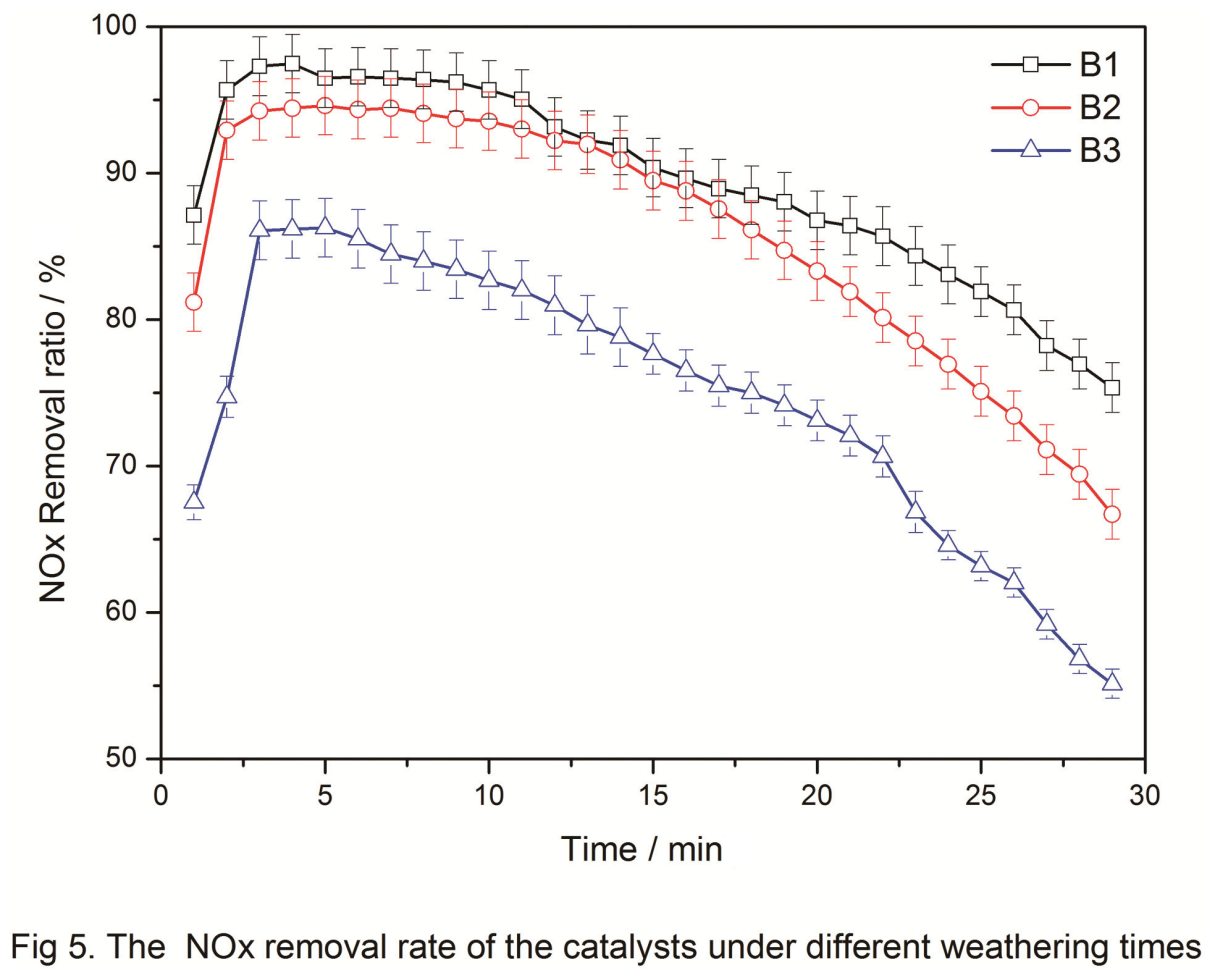
The NO_x_ removal rate of the catalysts under different weathering times.

About 1.5 g of B1, B2, and B3 was added to the flue gas simulation SCR reactor. The conditions of SCR were as follows: [NH_3_] = [O_2_]= 2[NO], 1000 ppm; reaction temperature, 353 K; and total N_2_ carrier gas flow, 800 mL/min. Mixed gases were added as mentioned in 2.2 when the temperature was stable. The inlet NO_*x*_ concentration was recorded when the outlet NO_*x*_ concentration stabilized. Then, NH_3_ was added and the outlet NO_*x*_ concentration was recorded every 1 min. Figure 5 shows the NO_*x*_ concentration of the three samples in reaction for different times.

Figure 5 shows that all three samples achieved optimum catalytic activity in reaction after 4-5 min from the beginning with 97% NO_*x*_ removal rate for B1, 95% for B2, and 85% for B3. Although the three samples almost simultaneously achieved the optimum catalytic activity, certain differences were observed. The catalytic activity remained unchanged after 24 and 48 h of weathering. However, with increased weathering time, the catalytic activity gradually decreased. Catalytic activity was also affected by weathering time. B1 reached the highest activity at 5 min but rapidly declined after only 5 min. After 20 min, the catalyst nearly showed no efficiency and the NO_*x*_ removal rate was below 80%.

In summary, long-term weathering destroyed the internal crystal structure of the catalyst, thereby reducing the catalytic activity. Thus, the best weathering time was 24 h.

### 3.5: Effect of GHSV

**Figure 6:**
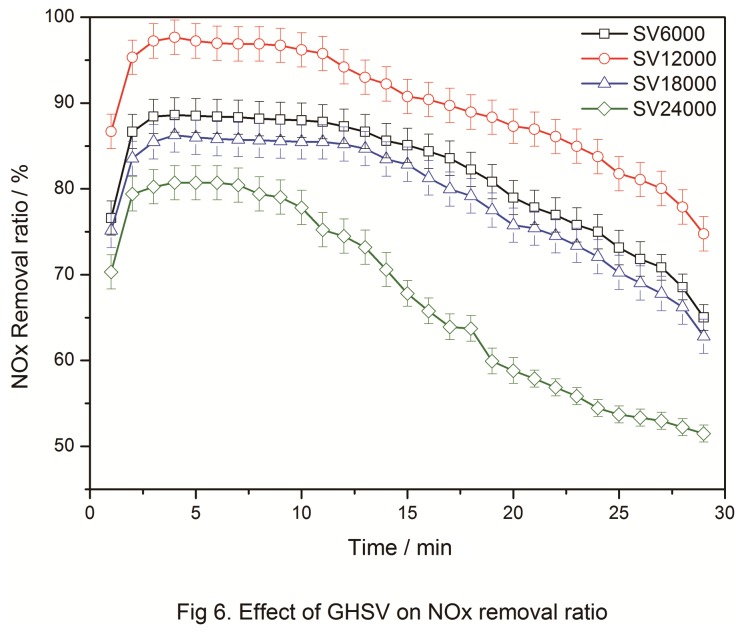
Effect of GHSV on NO_x_ removal.

GHSV is the gas speed during a reaction and affects various catalytic reactions differently. Low GHSV is more suitable for catalysts with a dense structure, whereas high GHSV is effective for catalysts with a loose structure. In this study, four kinds of GHSVs (6000 h^-1^, 12000 h^-1^, 18000 h^-1^, 24000 h^-1^) were used to evaluate the effects on NO_*x*_ removal at 353 K. The results are shown in Figure 6. The experimental conditions were as follows: [NH_3_] = [O_2_]= 2[NO], 1000 ppm; temperature, 353 K; carrier gas, N_2_; and catalyst amount, 1.5 g. Figure 6 shows that when GHSV = 12000 h-1, the NO_*x*_ removal reached 95% and the effective time was 10 min. With increased GHSV from 12000 h^-1^ to 24000 h^-1^, the removal rate caused by the catalyst significantly decreased. The removal rate also decreased when GHSV = 6000 h^-1^. Thus, the optimum GHSV was determined to be 12000 h^-1^. It showed that when the GHSV increased, the NOx removal efficiency would increase due to the decrease of out-diffusion, when the GHSV further increased, corresponding removal efficiency decreased a little, that might be that the increase of GHSV resulted in the more amount of gas and shortened the retention time for the gas, thus reaction could not finish completely. From this point of view, the proper GHSV was of great importance for NOx removal.

### 3.6: Effect of O_2_ concentration

O_2_ crucially affects SCR but minimally affects NH_3_ adsorption. Inomata [17] demonstrated that O_2_ cannot change the NH_3_ distribution on the catalyst in the low-temperature range but can facilitate O to capture the hydrogen in NH_3_. When the catalyst was pretreated with O_2_, the activity of the O group on the surface significantly increased. This phenomenon facilitated NO adsorption and increased thermal stability. The existence of O_2_ increased NO_2_ adsorption, and then O_2_ re-oxidized the active site of the catalyst surface and helped the catalyst to re-capture H in NH_3_ and initiate SCR.

**Figure 7:**
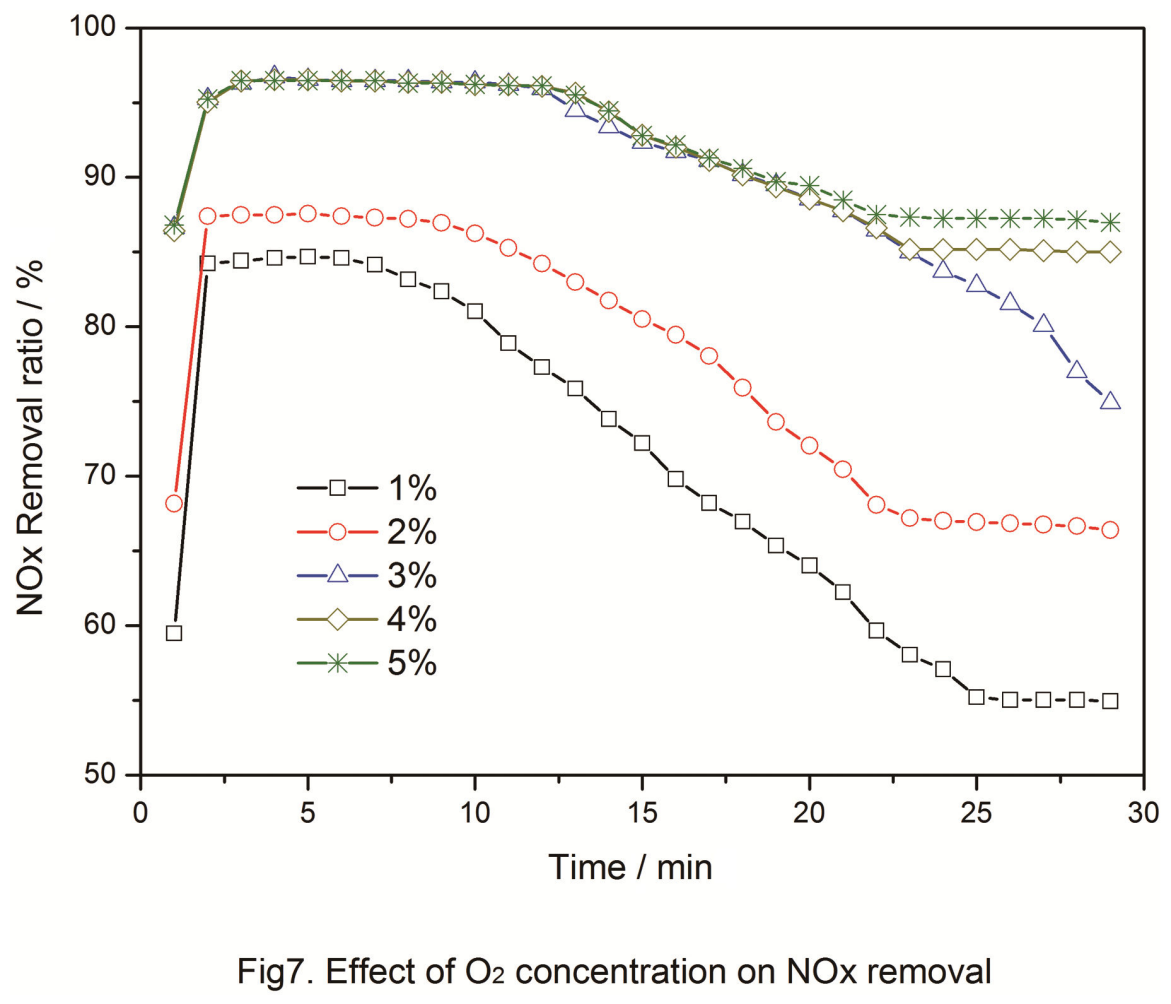
Effect of O_2_ Concentration on NO_x_ removal.

The effect of the O_2_ concentration in the catalyst on its performance was determined using five O_2_ concentrations (1%, 2%, 3%, 4%, and 5%). The corresponding results are shown in Figure 7. At a lower O_2_ concentration, the catalytic activity sharply decreased and did not recover to its original value. After O_2_ introduction, adsorbed O and lattice O quickly formed, and O_2_ was used for oxidation on the catalyst surface. Figure 7 shows that increased O_2_ concentration enhanced the catalytic activity. The maximum oxidation activity of 95.23% was obtained at 3% O_2_ concentration. The catalytic activity decreased with increased O_2_ concentration, but saturated concentrations did not significantly enhance the activity.

## Conclusion

The comparison among low-temperature Mn-containing SCR catalysts produced by co-precipitation, impregnation, and sol-gel in case of NOx removal efficiency demonstrated that the sol-gel method was determined to be the optimum choice with >90% NO_*x*_ removal rate. The optimum conditions for catalytic production were as follows: Mn/Ti ratio, 0.5; reaction temperature, 353 K; optimum manganese acetate concentration, 0.10 mol; under these conditions the removal efficiency could reach to about 95%. The produced catalyst were used for optimum reaction condition, and weathering time of 24 h; GHSV of 800 12000 h^-1^; and O_2_ concentration of 3% were proved to be the proper choice. The Mn-containing SCR catalysts produced by sol-gel methods showed good ability for NOx removal.
